# Confidence guides spontaneous cognitive offloading

**DOI:** 10.1186/s41235-019-0195-y

**Published:** 2019-12-02

**Authors:** Annika Boldt, Sam J. Gilbert

**Affiliations:** Institute of Cognitive Neuroscience, University College London, London, UK

**Keywords:** Cognitive offloading, Reminders, Delayed intentions, Prospective memory, Metacognition, Confidence, Metamemory

## Abstract

**Background:**

Cognitive offloading is the use of physical action to reduce the cognitive demands of a task. Everyday memory relies heavily on this practice; for example, when we write down to-be-remembered information or use diaries, alerts, and reminders to trigger delayed intentions. A key goal of recent research has been to investigate the processes that trigger cognitive offloading. This research has demonstrated that individuals decide whether or not to offload based on a potentially erroneous metacognitive evaluation of their mental abilities. Therefore, improving the accuracy of metacognitive evaluations may help to optimise offloading behaviour. However, previous studies typically measure participants’ use of an explicitly instructed offloading strategy, in contrast to everyday life where offloading strategies must often be generated spontaneously.

**Results:**

We administered a computer-based task requiring participants to remember delayed intentions. One group of participants was explicitly instructed on a method for setting external reminders; another was not. The latter group spontaneously set reminders but did so less often than the instructed group. Offloading improved performance in both groups. Crucially, metacognition (confidence in unaided memory ability) guided both instructed and spontaneous offloading: Participants in both groups set more reminders when they were less confident (regardless of actual memory ability).

**Conclusions:**

These results show that the link between metacognition and cognitive offloading holds even when offloading strategies need to be spontaneously generated. Thus, metacognitive interventions are potentially able to alter offloading behaviour, without requiring offloading strategies to be explicitly instructed.

## Significance

Psychologists usually study people’s ability to perform tasks without help from external tools and resources. Yet in everyday life we often extend our cognitive abilities using external resources. For example, if you want to remember an intention, you might write it down or use a diary, alert, or smartphone reminder. Recent studies have investigated how and when people decide to use *cognitive offloading* strategies such as this to support memory. A key finding is that people decide whether they need reminders based on how good they think their memory is, regardless of how good it objectively is. Therefore, if we can improve people’s insight into their memory, they might compensate more effectively by offloading when needed. However, a shortcoming of previous studies is that they usually measure whether participants use an offloading strategy that was explicitly explained to them. By contrast, offloading strategies in everyday life are usually adopted spontaneously. Here we administered a computer-based task requiring two groups of participants to remember delayed intentions. One group was told how to set external reminders but not the other. The latter group spontaneously developed a strategy for setting reminders but did so less often than the instructed group. Importantly, both groups were more likely to set reminders when they had lower confidence in their memory abilities. Therefore, both instructed and spontaneous offloading was guided by confidence. This suggests that improving individuals’ insight into their memory abilities can potentially optimise cognitive-offloading strategies, without those strategies needing to be explicitly instructed.

## Background

Imagine that your doctor gives you a prescription for medication to be taken every morning before breakfast. In order to remember to do so, you might choose to put the package of pills on your nightstand next to your alarm clock or place it in the cup holding your toothbrush. Alternatively, you might prefer to set an alarm on your smartphone or place a sticky note on your bathroom mirror. These are just a few examples of the type of reminders people use to support their memory for delayed intentions (i.e. *prospective memory*). Setting a reminder to prompt a delayed intention is an example of *cognitive offloading*: the use of physical action to reduce the cognitive demands of a task (Risko & Gilbert, 2016). Recent research has begun to investigate how and when people decide to use cognitive offloading as a strategy to support their prospective memory (Cherkaoui & Gilbert, 2017; Gilbert, 2015a, 2015b; Gilbert et al., in press; Redshaw, Vandersee, Bulley, & Gilbert, 2018). Similarly, cognitive offloading has been studied in the context of problem solving (Chu & Kita, 2011), learning (Costa et al., 2011), mental rotation (Dunn & Risko, 2016), and retrospective memory (Finley, Naaz, & Goh, 2018; Henkel, 2014; Risko & Dunn, 2015; Soares & Storm, 2018; Storm & Stone, 2015). One goal of this research is to understand the mechanisms by which individuals decide whether or not to engage in cognitive offloading. This may ultimately lead to interventions that can optimise individuals’ use of external resources to support cognition. However, the mechanisms that trigger cognitive offloading are not well understood at present. Here, we investigate these mechanisms by examining the circumstances under which participants decide to use external reminders to support their memory for delayed intentions. In particular, we examine whether these circumstances are similar when an offloading strategy is explicitly instructed compared with a situation where it needs to be spontaneously generated.

### Measuring cognitive offloading

**Fig. 1:**
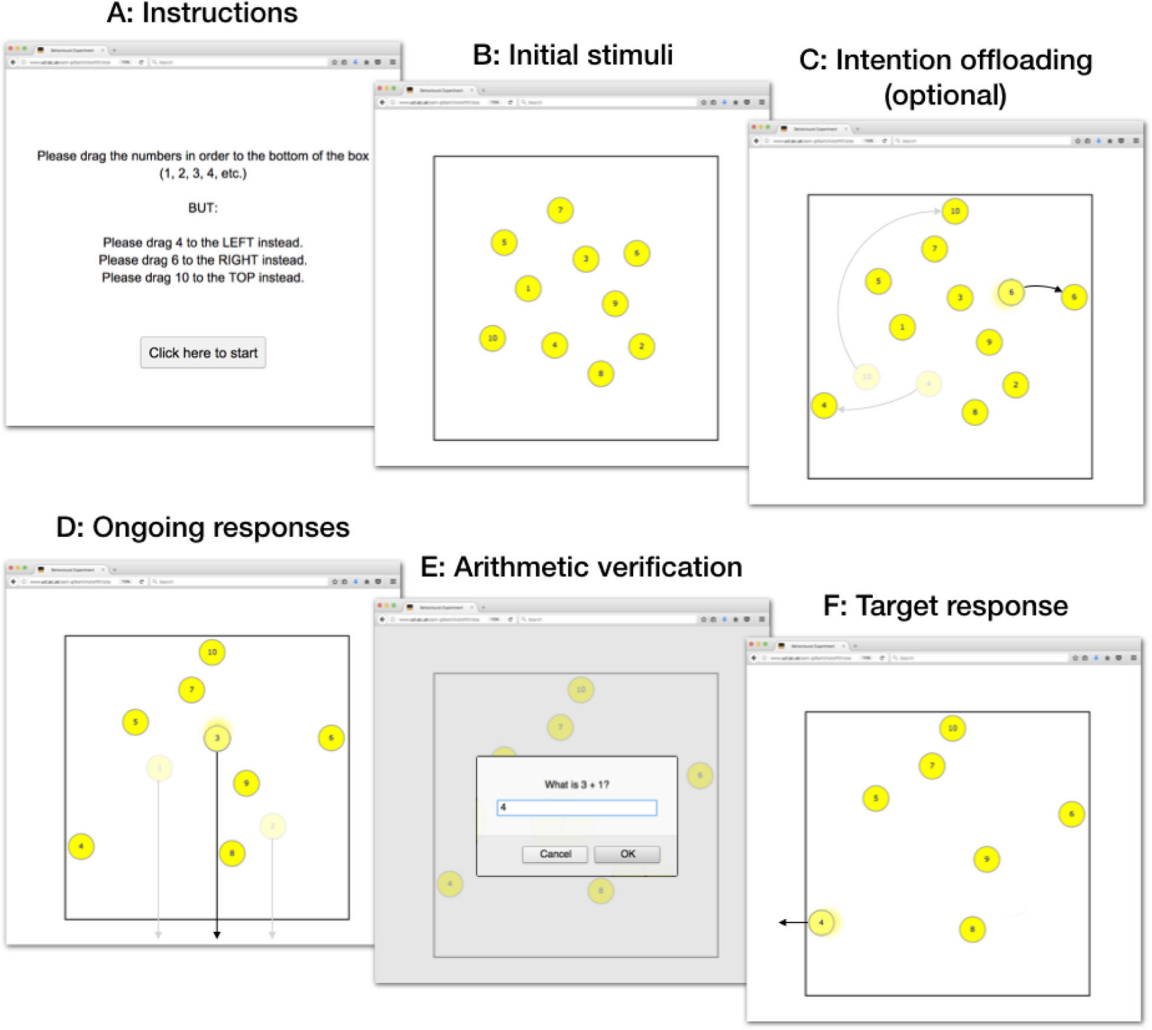
Schematic illustration of the intention offloading task from a previous study (Gilbert, 2015a). Prior to each trial, participants were presented with an instruction (**a**), informing them about three target circles. They were then faced with 10, randomly arranged circles (**b**). In some phases of the task, people were free to set reminders by placing the target circles close to their instructed border (**c**). The main task was to move the circles out of the bottom of the square in numeric order (**d**). Before the first target circle was reached, an arithmetic verification task was presented (**e**). After submitting a response, participants continued with the next circle, in this example by moving the first target circle off the square (**f**). The trial ended once all circles had been removed

Several laboratory paradigms have recently been developed to measure cognitive offloading in experimental conditions. For example, in Gilbert (2015a), participants completed an online task in which they used their mouse to drag 10 circles to the bottom of the screen in numerical order (see Fig. 1 for a schematic illustration of this task). Prior to each trial, participants were instructed that either one or three of these circles (*target* circles) had to be moved to the top, left, or right border instead of the bottom (Fig. 1a). These instructions constituted the delayed intentions. In some conditions, participants had to rely entirely on their own internal memory to remember these intentions. In others, however, participants were allowed to set reminders: They were told that they could move the three target circles close to their instructed side at the beginning of the trial to remind themselves of the intention when they eventually reached the target circles in the numerical sequence (Fig. 1c). The use of such an explicitly instructed cognitive-offloading strategy improved performance. Furthermore, participants offloaded more often when the task was more difficult; for example, when there were three intentions to remember rather than one, or when they were asked a distracting arithmetic question between encoding an intention and executing it (Gilbert, 2015a).

### Metacognitive guidance of cognitive offloading

These results suggest that participants decide whether or not to offload based on a metacognitive evaluation of the difficulty of a task. This hypothesis was tested in a follow-up study by Gilbert (2015b). In the first phase, participants performed the above described circle-dragging task without the ability to set reminders. They also reported their metacognitive confidence in their ability to perform the task. In the second phase, participants performed the same task, this time with the option to set reminders if they wished. Unaided performance in Phase 1 negatively predicted offloading: People who remembered fewer target circles in the first phase were more likely to set reminders in the second. Independently of this, participants’ metacognition (their confidence in their unaided abilities) also predicted offloading. The less confident people were that they would fulfil the delayed intentions on their own, the more likely they were to set reminders (see also Cherkaoui & Gilbert, 2017; Risko & Gilbert, 2016). This effect was present even if confidence was measured in a separate perceptual task that was unrelated to participants’ mnemonic accuracy (Gilbert, 2015b, Experiment 2). These findings indicate that cognitive offloading can be guided by metacognitive beliefs, independently of individuals’ objective unaided ability.

Relatedly, Dunn and Risko (2016) found evidence that perceived difficulty influenced the decision to offload. The authors used a reading task in which the text was sometimes tilted to one side. Participants could offload the demand for mental rotation by physically tilting their head. They tended to do so more in a condition they perceived as more difficult, not necessarily the condition that was objectively more difficult. A similar notion has been raised in the skill-acquisition literature: Touron and Hertzog (2004) for instance found that age differences in strategy use in a noun-pair task can be attributed to a difference in confidence between the age groups, not a difference in skill. More specifically, they observed that older participants took longer to switch from a scanning strategy (using a lookup table) to a retrieval strategy (relying on their own memory) despite comparable retrieval performance measured during separate memory-probe trials. A recent review by Hertzog and Dunlosky (2011) suggested that metacognition regulates the use of compensatory strategies in a range of domains and that this mechanism can thus support the independence of older adults in everyday life. For example, using a structural-equation modelling approach, Simon and Schmitter-Edgecombe (2016) investigated the use of compensatory memory strategies in healthy adults aged 55 years or older. They found support for the hypothesis that memory self-efficacy—beliefs people hold about their own memory performance—mediates the relationship between objective memory performance and the use of compensatory strategies. More specifically, participants who reported higher confidence in their memory skills also reported a more infrequent use of compensatory memory strategies (see also de Frias, Dixon, & Backman, 2003; McDougall, 1996).

### Instructed and spontaneous offloading

The evidence reviewed above indicates a role of metacognitive processes in triggering cognitive offloading in a variety of domains (see also Risko & Gilbert, 2016). This suggests that metacognitive beliefs could be a target for interventions which could improve individuals’ adaptive use of external cognitive resources. For example, prospective-memory abilities might be improved not only by directly retraining the memory and executive functions components that allow the fulfilment of delayed intentions (e.g. Fish, Wilson, & Manly, 2010; Raskin & Sohlberg, 1996) but also by improving people’s insight into their own memory performance to then compensate by cognitively offloading where needed (for an ongoing trial see also Fleming et al., 2017). Population ageing has made investigation into compensatory strategies even more important, as efficient reminder-setting can play a core role in supporting older people to live independently (Phillips, Henry, & Martin, 2008). However, a crucial limitation shared by almost all research into cognitive offloading of delayed intentions is the fact that participants are usually explicitly told about the possibility of setting reminders, both in the case of laboratory work with healthy adults (e.g. Gilbert, 2015a), as well as clinical trials with neurological patients (e.g. Fleming et al., 2017) or older adults (e.g. Einstein & McDaniel, 1990). In these explicit offloading paradigms, participants are usually told about a compensatory strategy that can be used to support remembering delayed intentions and are then given a choice of whether or not to use it. However, it remains unclear whether similar use of reminders can be replicated in a setting in which participants are not explicitly told about the existence of a compensatory strategy. This would be more akin to everyday life, where offloading strategies typically need to be spontaneously generated rather than being explicitly instructed by an experimenter.

In the present study we investigate whether confidence regulates cognitive offloading, not only when the offloading strategy is explicitly instructed, but also in a more naturalistic setting where it has to be self-generated. Although previous research has shown that individuals with lower confidence in their abilities offload more often (Gilbert, 2015b), this might be dependent on explicitly describing an offloading strategy. Alternatively, individuals who spontaneously offload intentions without being explicitly informed about this strategy might be already highly able and motivated, leading to a ‘rich get richer’ effect rather than the compensatory effect found in previous studies. Critically, we chose to measure cognitive offloading directly rather than relying on self-report methods commonly used (e.g. Simon & Schmitter-Edgecombe, 2016), which can be subject to potential biases. We addressed these questions in a variant of the online experiment described above in which participants had to drag circles to the bottom of a square in a certain order, while keeping several delayed intentions in mind (Gilbert, 2015b). Our experiment was comprised of two main parts: During Phase 1 we measured baseline fulfilment of delayed intentions, whereas in Phase 2 we allowed intention offloading and measured both the uptake of this compensatory strategy as well as the extent to which it improved performance. Participants were randomly assigned to one of two groups, only one of which received explicit instructions about the offloading strategy. The key methodological challenge of our study was to develop a paradigm that not only allowed participants to spontaneously develop offloading strategies, but also ensured that they would not feel that they were ‘misbehaving’ when using such a compensatory mechanism. We therefore included a cover story (‘charging’ of the circles prior to each trial by moving them) that taught participants that it was acceptable to manipulate the position of the circles on the screen.

## Methods

### Participants

A total of 435 participants were recruited through the Amazon Mechanical Turk (MTurk) website (http://www.mturk.com). The sample size chosen gave us sufficient power to detect whether there was a small effect (*d* = 0.2) of externalising in the Spontaneous Offloading Group. Three hundred and sixty-three datasets were used in the final analysis (see the ‘Results’ section for the exclusion criteria). While many laboratory studies are based on highly homogeneous samples of undergraduate volunteers, MTurk has the advantage that it allows researchers access to more representative samples, thereby often increasing generalisability to the general public. However, we decided to restrict recruitment to participants based in the USA to ensure the required level of English proficiency. Of the resulting sample, 178 were men, 184 women, and one reported not identifying with binary gender categories. Participants were on average 34.1 years old (minimum = 18 years; maximum = 73 years). We paid all participants US$5 for their participation. The average of the duration of the task was 34 min (minimum = 13 min; maximum = 5 h 18 min). Ethical approval for this study was received from the local Ethics Committee and informed consent was obtained from all participants prior to the study. Participants were randomly assigned to one of two groups: One hundred and eighty-eight formed the Instructed Offloading Group and 175 the Spontaneous Offloading Group.

### Task and procedures

We used a web-based task that allowed measurement of the fulfilment of delayed intentions (Gilbert, 2015a, 2015b), using Google Web Toolkit (GWT). The task was designed to be completed on people’s own computers in a web browser using a computer mouse, trackpad, or touch screen. On each trial and similar to Gilbert (2015a), 10 yellow circles appeared randomly positioned within a box (Fig. 1a). Participants’ task was to move the circles to the bottom of the box, where they would disappear. Each circle had to be moved in turn, according to the order indicated by its label (1 to 10). After the last circle disappeared, the screen cleared and the next trial began. A demonstration of this basic task can be found at https://www.ucl.ac.uk/sam-gilbert/demos/circleDemo.html.

Prior to each trial, participants were informed that there were three ‘special circles’: circles that had to be moved to the left, top, or right border of the square instead of the bottom. These instructions constituted the delayed intentions, meaning that participants had to delay fulfilling them until it was the turn of the respective circles to be moved. In this task, there was no error feedback (as opposed to Gilbert, 2015a, 2015b) and both target and non-target circles could be moved to any of the four borders of the square.

Following the protocol from Gilbert (2015a, 2015b), participants were furthermore asked to solve a distracting arithmetic question during each trial (Fig. 1e). This was done to make the task more difficult and to thus avoid that memory performance (accuracy in moving the target circles to their correct borders) would level at ceiling. The question appeared on screen at a random position in the trial before they removed the first target circle from the screen.

Participants first completed seven practice trials which incrementally explained different features of the task. In our task, offloading meant moving the three ‘special circles’ close to their unique boundaries (e.g. to move circle 7 close to the top boundary) so that there is no need to remember where it has to be moved when the time comes to make the seventh move. To measure baseline unaided memory performance, participants then completed a block of 10 trials where offloading was not possible and participants thus had to rely solely on their own memory. This was done by fixing the position of all circles on the screen apart from the next in the sequence, so that the eventual target circles could not be moved into a reminder position. This part constitutes Phase 1 of the experiment. Each of the 10 trials in this phase included moving three target and seven non-target circles—in other words 30 data points contributed to each participants’ calculation of this baseline measure. During this block, participants had to rely solely on their internal memory to remember the delayed intentions of moving the target circles to the other borders. During the entire experiment, we recorded not only the start and end positions of any dragging movement, but also the continuous movement data of the cursor. However, due to insufficient data transfer speed a large proportion of these data was not recorded and will, therefore, not be reported further.

**Fig. 2:**
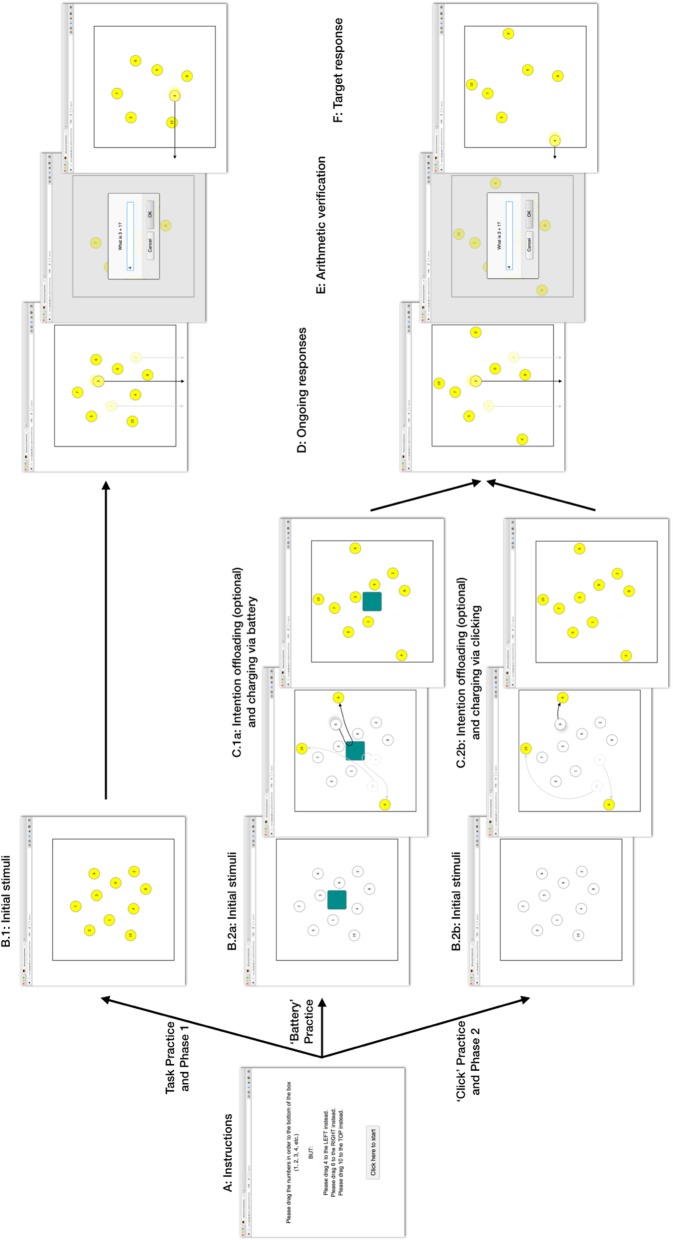
Schematic illustration of the intention offloading task. The main task was to move the circles out of the square in their correct order (1 to 10). Prior to each trial, participants were presented with an instruction (A), informing them about three target circles. They were then faced with 10, randomly arranged circles (B). During initial task practice and Phase 1, all initial stimuli appeared on the screen already charged (i.e. filled yellow) and intention offloading was not possible. Prior to the second phase, intention offloading was made possible. A brief practice phase ensured that participants got used to the idea of freely moving the circles around prior to each trial. A compulsory step of ‘charging’ the circles was, therefore, included. Participants were presented with the 10 circles randomly arranged in addition to a ‘battery’. Participants were instructed that they had to move all circles over the battery to ‘charge’ them, colouring them yellow, before continuing with the task (B.2a; ‘Battery’ Practice). During this step, they were allowed to set reminders. Note that the three example paths of the target circles (C.1a) led across the battery, but that participants could just as well choose to move the circles without charging them, as well as charge them without cognitively offloading. These steps were slightly altered during the last practice trial that led into Phase 2 (‘Click’ Practice): the ‘uncharged’ circles appeared without the battery (B.2b). Before any circles could be moved out of the square, each had to be ‘charged’ by clicking on it or moving it (C.2b). The trial started and the default border over which circles had to be dragged to remove them was the bottom border (D). Before the first target circle was reached an arithmetic verification task was presented (E). After submitting a response, participants continued with the next circle, in this example by moving the first target circle off the square (F). The trial ended once all circles had been removed

Before the second phase of the experiment, during which offloading was allowed, we included another brief practice block of 4 trials. The purpose of these practice trials was to make participants aware that they could adjust the position of the circles before beginning to drag them to the bottom of the box. Allowing participants to freely rearrange the circles on screen meant that they could set reminders by moving the target circles closer to the respective borders of the square (see Fig. 2C). This rearrangement meant that they no longer had to rely on their own memory to fulfil the delayed intentions. However, since half of the participants had to self-generate the offloading strategy, we could not explicitly tell them that the circles could be moved freely as it could have potentially made the reminder strategy too obvious. At the same time, we also had to ensure that participants would not feel like they were misbehaving when they offloaded. We used a cover study according to which the circles had to be ‘charged’. More specifically, every trial would begin with all 10 circles displayed in white (see Fig. 2). People had to move every circle over a centrally placed ‘battery’ to activate them (i.e. colour them yellow). Only after all circles had been activated were they able to continue dragging the circles in sequential order off the square. The battery was removed during the last practice trial and participants were instructed that they could now charge the circles simply by clicking on them. However, they were told that they were still free to move the circles around. This change ensured that any dragging movement we recorded (e.g. dragging a target circle next to its instructed location) was not simply an attempt to ‘charge’ the circle.

Only the Instructed Offloading Group was explicitly told about the intention offloading strategy during the second phase of the experiment. More specifically, they saw an additional screen reading ‘[ … ] you will now be able to rearrange the circles on the screen if you like. Some people find it helpful to drag the special circles near to the edge of the box to help them remember. [ … ] You should feel free to use this strategy if you like, but it’s up to you’ (see Additional file 1 for full instructions). The conditions that the two groups performed did not differ in any other regard.

In addition to the delayed-intentions task, we measured metacognitive beliefs. Participants were asked to rate their confidence in their ability to correctly execute intentions by moving a slider ranging from 0% (none of the target circles correctly remembered) to 100% (all target circles correctly remembered) before and after each phase. This procedure resulted in two prospective and two retrospective confidence estimates per participant. Here, we refer to these measures as *Phase 1 prediction*, *Phase 1 postdiction*, *Phase 2 prediction*, and *Phase 2 postdiction*.

After completion of all trials, participants were asked to fill in two short questionnaires: The first questionnaire was the cognitive confidence sub-scale from the short version of the Metacognitions Questionnaire (MCQ-30; Wells & Cartwright-Hatton, 2004). Including this psychometric tool allowed us to validate the confidence judgements. Secondly, we included the 10-item sub-scale for conscientiousness retrieved from http://ipip.ori.org. This assessment was included for a separate research question. Conscientiousness scores did not show any systematic relationship with our other measures; we will, therefore, not report these measurements any further. Participants were furthermore asked whether they used any and if so which internal or external strategies and why they made use of such strategies. They were also given the opportunity to comment on the study.

### Data analysis

All data analyses were conducted using *R* version 3.4.1. Bayes Factors were calculated using the *BayesFactor* package (Rouder, Speckman, Sun, Morey, & Iverson, 2009) with the package’s default settings (scaled JZS Bayes Factor with a Cauchy prior and *r* = 0.707). We report Bayes Factors for all *t* tests. *BF*_*10*_ indicates a Bayes Factor favouring the alternative hypothesis, whereas *BF*_*01*_ indicates a Bayes Factor favouring the Null hypothesis. Our analyses focussed on two key dependent variables: *target accuracy* and *externalizing proportion*. Target accuracy was defined as the proportion of target circles that was moved to the instructed boundary rather than to the bottom of the square. The externalising proportion is a measure of intention offloading (as in Gilbert, 2015a, 2015b): We calculated the difference between the proportion of target circles moved before their turn in the numerical sequence, minus the proportion of non-target circles moved before their turn. The rationale for this measure is that participants occasionally move circles before their turn in the sequence simply due to picking up the wrong circle by accident. This would not constitute offloading. By subtracting the likelihood of moving a non-target circle before its turn in the sequence from the equivalent number for target circles we can obtain a measure of offloading behaviour that is selectively directed towards target circles, corrected for any general tendency to accidentally select the wrong circle. We only included movements that exceeded 100 pixels on screen when calculating this measure to avoid including accidental displacements of circles during the ‘charging’ phase of the trials. To form the externalising proportion, we then subtracted the proportion of offloaded non-targets from the proportion of offloaded targets.

To address the second key question of this study—whether spontaneous offloading is guided by metacognitive beliefs—we conducted a path analysis similar to the one used in Gilbert (2015b). This analysis was based on several linear regressions, which were fitted separately for each dependent variable using all antecedent measures as predictors. This was done independently for the two groups. The path weights reported reflect standardised beta coefficients and significance testing reflects the significance of each predictor in the respective linear regression. This analysis can be seen as a form of structural equation modelling in which variables were directly observed measures rather than latent factors (Garson, 2012). We included the following variables in this path analysis: Phase 1 prediction, Phase 1 performance, Phase 2 prediction, Phase 2 offloading behaviour, and Phase 2 performance (listed according to their sequential order in the experiment). For simplicity and in accordance with the analysis performed by Gilbert (2015b), the two postdiction measures were not included in the path analysis. In a second step, we then compared whether there was any difference in the path weights between the two groups using an Analysis of Covariance (ANCOVA). More specifically, we included ‘group’ as an additional factor in each regression analysis and then used a model comparison approach to assess whether the resulting slopes differed between the two groups, which would have been reflected in a significant interaction between the group factor and any other predictor.

## Results

### Overall task performance

Of the 435 participants, 72 were excluded. We excluded datasets from participants who took part in the study more than once (*N* = 3), had taken part in similar offloading experiments in our laboratory (*N* = 43), had low arithmetic accuracy (*N* = 3 had scores lower than 80%, whereas average accuracy was high at 99.3%), or reported using task-external memory aids, such as note taking (*N* = 23). The final sample consisted of 363 participants. Results remained similar even when the 43 participants who had taken part in similar offloading studies were included in the sample. The overall mean retention interval (i.e. the time interval from the start of the trial to the first target circle) was 21 s and false alarms (i.e. incorrectly moving non-target circles to the top, left or right border) were rare at an overall rate of 3.4%. This is comparable to our previous studies using this task (Cherkaoui & Gilbert, 2017; Gilbert, 2015a, 2015b).

**Fig. 3:**
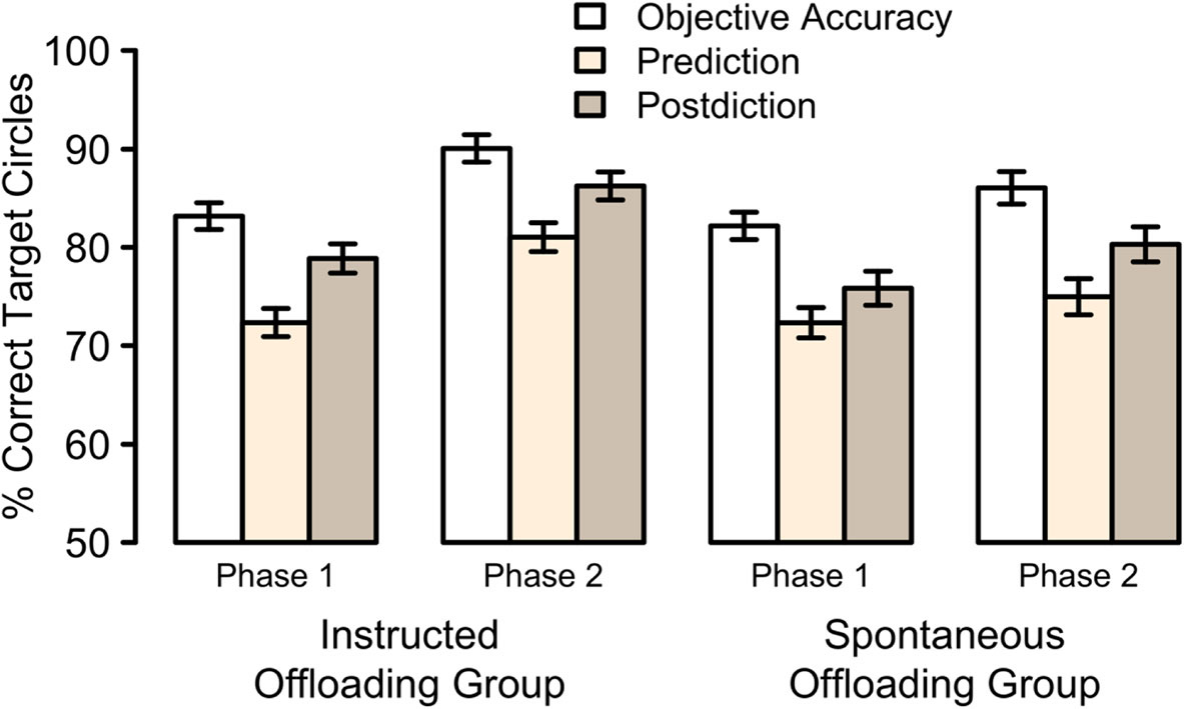
Percentage of target circles that were moved to the correct border (top/left/right) as a function of group, phase of the experiment, as well type of judgement (objective accuracy, prediction, and postdiction). All error bars are standard errors of the mean

Participants had an average hit rate of 85.4% (see the white bars in Fig. 3). Performance was slightly higher in the Instructed Offloading Group (87.2%) compared to the Spontaneous Offloading Group (83.8%). When submitted to a mixed-design ANOVA with both Group and Phase as factors, this difference did not reach significance, *F* (1,361) = 1.8, *p* = 0.18, η^2^_G_ = 0.004. We found a reliable difference between phases, *F* (1,361) = 39.1, *p* < 0.001, η^2^_G_ = 0.019, reflecting that on average, hit rates were higher for Phase 2 (88.1%) compared to Phase 1 (82.7%), replicating previous findings from our laboratory (e.g. Gilbert, 2015a). Posthoc *t* tests revealed that this difference was reliable for both the Instructed Offloading Group, *t* (187) = 5.7, *p* < 0.001, *BF*_*10*_ = 206,357.1, as well as for the Spontaneous Offloading Group, *t* (174) = 3.1, *p* < 0.01, *BF*_*10*_ = 8.4. The interaction between both factors was not found to be reliable at our chosen α level of 0.05, *F* (1,361) = 3.0, *p* = 0.08, η^2^_G_ = 0.001. We furthermore tested whether there was any difference between groups in Phase 1, that is when both groups faced the exact same task and instructions and without excluding any participants. A Welsh two-sample *t* test revealed no reliable difference between the groups, *t* < 1. To further quantify the support for the Null hypothesis, we used a Bayesian *t* test, which yielded a *BF*_*01*_ of 7.6 in favour of the hypothesis that both groups can best be described by the same distribution during this initial phase of our study. In Phase 2, the Instructed Offloading Group had higher hit rates (90.5%) compared to the Spontaneous Offloading Group (86.0%). However, this difference did not reach significance at our chosen α level of 0.05, *t* (346.07) = 1.9, *p* = 0.06, *BF*_*01*_ = 1.6. Taken together, our analyses revealed that allowing participants to offload (Phase 2) increased performance. Our analyses did not reveal any clear differences between the two groups. However, further analyses will test whether there were any differences in terms of offloading behaviour.

### Basic metacognitive accuracy

A key dependent measure in this study was the confidence ratings given by participants prior to and after the two main phases of the experiment. As expected, these prediction and postdiction measures correlated significantly and negatively with people’s average MCQ scores, *r*s < − 0.34, *p*s < 0.001, suggesting that these ratings did reflect metacognitive belief. Figure 3 depicts the confidence averages as a function of group, phase, and type of judgement (beige and brown bars) together with their objective accuracy (white bars). Overall, participants were underconfident, meaning that both their overall prediction and postdiction estimates were significantly lower than their average accuracy, *t*s > = 5.6, *p*s < 0.001. We then submitted the confidence data to a mixed-design ANOVA with Group, Phase, and Confidence Type (prediction or postdiction) as factors. Prediction was found to be more pessimistic than postdiction, *M*_*p*_ = 75%, *M*_*r*_ = 80%, *F* (1,361) = 63.8, *p* < 0.001, η^2^_G_ = 0.015. Confidence estimates during Phase 2 of the experiment were higher than during Phase 1, *M*_*1*_ = 75%, *M*_*2*_ = 81%, *F* (1,361) = 58.8, *p* < 0.001, η^2^_G_ = 0.019. Both of these main effects constitute replications of previous findings from our group (Gilbert, 2015b). Overall confidence was higher in the Instructed Offloading Group, *M*_*I*_ = 80%, *M*_*S*_ = 76%, but this effect did not reach significance at our chosen α level of 0.05, *F* (1,361) = 3.8, *p* = 0.052, η^2^_G_ = 0.008. We furthermore found a reliable Group by Phase interaction, *F* (1,361) = 8.5, *p* < 0.01, η^2^_G_ = 0.003, meaning there was a larger increase in confidence across phases for the Instructed Offloading Group (*M* = 8%), compared to the Spontaneous Offloading Group (*M* = 4%). None of the other two-way interactions nor the three-way interaction were significant, *F*s < 2.1, *p*s > 0.15, η^2^_G_s < 0.0003. Given that the group difference was found to be non-significant at *p* = 0.052, we calculated Bayes Factors (against an additive Null model of the grand mean and the only random factor, Participant) for every possible combination of main and/or interaction effects to then select the best fitting model using a model-comparison approach. A model with only two main effects (Phase and Confidence Type) and the Group by Phase interaction resulted in a higher Bayes Factor, *BF* = 1.9e + 27, than a model which also included a main effect of Group, *BF* = 1.5e + 27. We thus conclude that there was no reliable difference between the two groups. Taken together, these findings suggest that participants were more confident in the second half of the experiment with an overall tendency towards underconfidence.

### Instructed and spontaneous reminder-setting

The first key question of this study was whether we would find reminder-setting even in the group that was not explicitly instructed to use this strategy (Spontaneous Offloading Group). In a first-pass analysis, we used one-sample *t* tests to determine whether both groups would show use of this strategy. Only data from Phase 2 of the experiment during which offloading was possible was, therefore, included in this analysis. The Instructed Offloading Group had an average externalising proportion of 53.1%, which was reliably different from zero, *t* (187) = 17.3, *p* < 0.001, *BF*_*10*_ = 2.4e + 37. Interestingly, the same held for the Spontaneous Offloading Group, which had a considerably lower average externalising proportion of only 38.9%, but which was still significantly different from zero, *t* (174) = 12.1, *p* < 0.001, *BF*_*10*_ = 3.9e + 21. The groups differed significantly in their use of reminders, *t* (358.3) = 3.2, *p* < 0.01, *BF*_*10*_ = 15.0. The distributions of offloading proportions across participants were bimodal, suggesting that most participants chose one strategy and ‘stuck with it’ for the entire experiment, though some people showed a mixture of both offloading and not offloading (see also Additional file 1: Figure S1). However, it is unlikely that such bimodality would have rendered our results invalid given that the *t* test has been found to be highly robust against such violations, especially for larger sample sizes (Lumley, Diehr, Emerson, & Chen, 2002). Nevertheless, when submitted to non-parametric alternative tests, the results remained significant (see Additional file 1). These findings show that intention offloading can be observed in our task, even when the strategy has to be spontaneously generated.

**Fig. 4:**
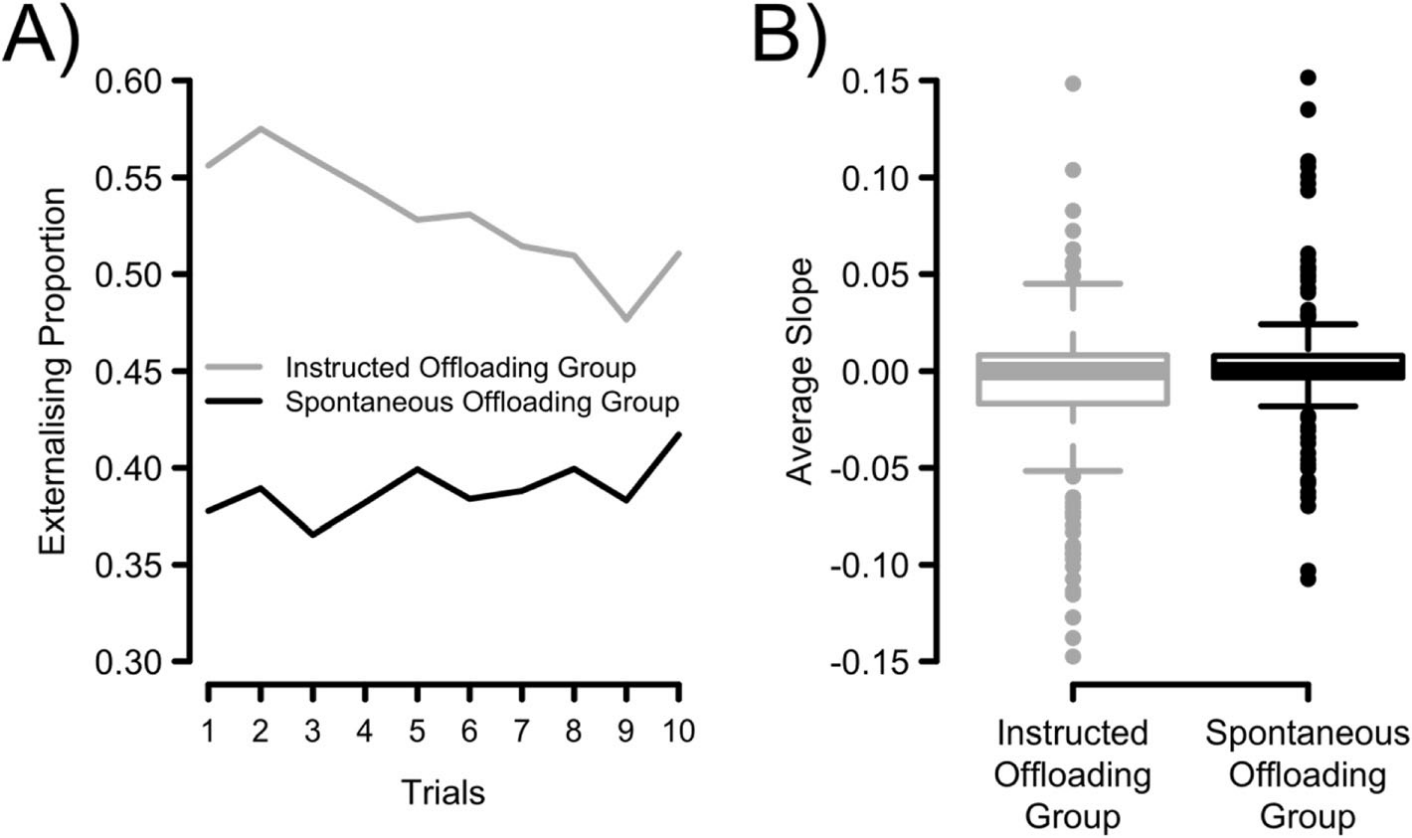
**a** Externalising proportion (proportion of non-target trials moved minus proportion of target trials moved) across trials. **b** Group-wise distributions of individual slopes from a regression model in which trial predicts the externalising proportion. The boxplots reflect the interquartile range (IQR) together with the median. The whiskers span from the first quartile minus 1.5 times the IQR to the third quartile plus 1.5 times the IQR. Data points outside this inner fence are defined as outliers and are shown as individual data points

In a post-hoc analysis we explored how strategic reminder-setting varied across trials in the two groups. Figure 4a depicts the externalising proportions over the course of 10 trials that comprised Phase 2 of the experiment. From visual inspection, it can be concluded that both groups varied qualitatively in their use of reminders over time: While the Instructed Offloading Group decreased in their average externalising proportion over time, the Spontaneous Offloading Group remained relatively constant and showed—if anything—a slight upward trend in their average externalising proportion. The two groups differed significantly in their externalising proportions for the first eight of the 10 trials (both at *p* < 0.05 and when tested using a permutation test, e.g. Maris & Oostenveld, 2007) but not the last two trials (only when tested using a permutation test, but not at *p* < 0.05). To further quantify this effect and to test whether the two groups really differed in their trial-by-trial dynamics, we fitted individual regression lines to each participant’s 10 data points (predicting the externalising proportion from the trial number). When averaged, the resulting slopes were indeed negative and significantly different from zero for the Instructed Offloading Group (*b* = − 0.009; 0.9% decrease in the externalising proportion per trial; *t* (187) = 2.9, *p* < 0.01, *BF*_*10*_ = 4.5) and positive but not significantly different from zero for the Spontaneous Offloading Group (*b* = 0.003; 0.3% increase in the externalising proportion per trial; *t* (174) = 1.1, *p* = 0.28, *BF*_*01*_ = 6.7). The difference in slopes between groups was significant, *t* (359.16) = 2.9, *p* < 0.01, *BF*_*10*_ = 5.7. However, despite this being a consistent and reliable difference, it should be noted that the slope distributions for the two groups largely overlapped (see Fig. 4b): In both groups, there were participants that increased or decreased in their use of reminders, but for most participants the externalising proportions remained stable across trials (slopes close to zero).

### Do metacognitive beliefs guide the use of compensatory strategies?

**Fig. 5:**
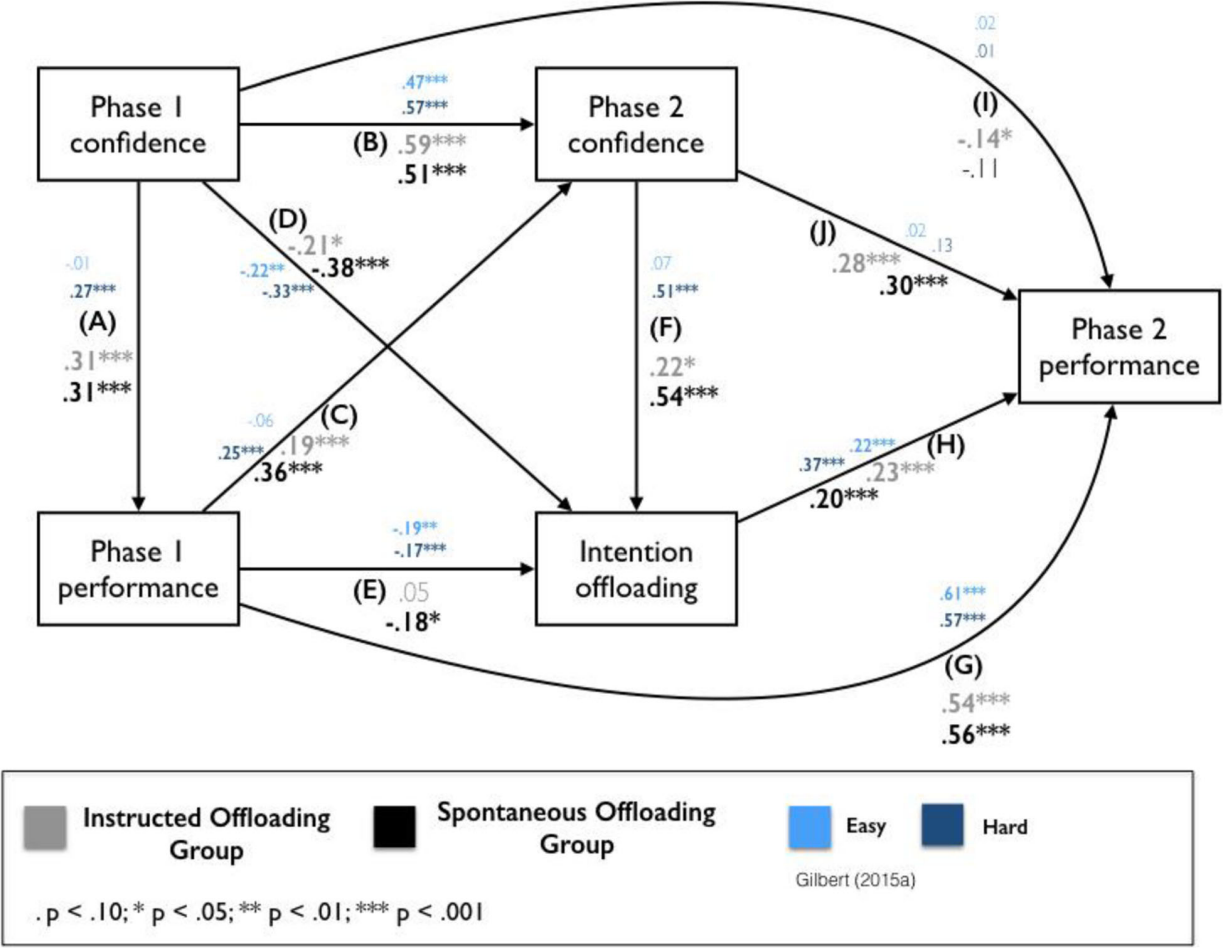
Path analysis model of the relationships between different measures recorded during the study. Standardised beta weights for the Instructed Offloading Group are shown in grey. Weights for the Spontaneous Offloading Group are shown in black. Results for Gilbert (2015b) are shown in blue. Boldface indicates a reliable path weight

Having established that spontaneous reminder-setting occurs in our experimental task and that people hold metacognitive beliefs into their ability to fulfil delayed intentions, the second key question that remains is whether such insight guides the decision to offload. To address this question, we conducted a path-diagram analysis that allowed us to test for the hypothesis that people tend to offload more when they have low confidence while controlling for a range of other, related variables. Figure 5 shows the resulting path diagram with path weights indicated for the two groups separately (grey for the Instructed Offloading Group, black for the Spontaneous Offloading Group; see also Additional file 1 for the raw correlations) as well as the results from Gilbert (2015b) in blue to allow for a direct comparison. The key finding from this analysis is the reliable negative influence of confidence during Phase 1 on intention offloading during Phase 2 (D). This was the case not only for the Instructed Offloading Group, β = − 0.21, *p* = 0.03, replicating the findings by Gilbert (2015b), but also for the Spontaneous Offloading Group, β = − 0.38, *p* < 0.001. More specifically this means that the lower people’s confidence was in their unaided ability to perform the task, the more likely they offloaded by setting reminders instead of solely relying on their own internal memory. Confidence was measured after people had completed a number of practice trials to familiarise themselves with the task to allow for accurate insight. Importantly, path weight (D) was calculated by fitting linear regressions to participants’ data using both Phase 1 confidence and Phase 1 performance as predictors. In other words, the effect of confidence on offloading was present independent of how well people actually performed. Interestingly, the influence of prediction during Phase 2 on intention offloading during Phase 2 (F) is reliably positive for both the Instructed Offloading Group, β = 0.22, *p* = 0.03, as well as the Spontaneous Offloading Group, β = 0.54, *p* < 0.001. This finding replicates Gilbert (2015b) and makes sense if we consider that this judgement was taken directly after participants had been introduced to the offloading strategy (explicitly or by generating it themselves). Participants who made a conscious decision to use the strategy during the second phase of the experiment might have consequently been more confident in their performance prediction. And rightly so, as intention offloading did positively predict performance during Phase 2 (H): The more people made use of this strategy, the more target circles they ended up moving to the correct border. This finding was true for both groups (Instructed Offloading Group: β = 0.23, *p* < 0.001; Spontaneous Offloading Group: β = 0.20, *p* < 0.001).

We considered two possible patterns describing the relationship between objective unaided memory performance and externalising behaviour (E): On the one hand, participants with lower unaided ability might offload more often as a compensatory strategy. This would predict a negative relationship between Phase 1 performance and Intention Offloading. Alternatively, participants who are particularly motivated and able might be more likely to make use of strategies (‘rich get richer’ effect), which would predict a positive relationship between Phase 1 performance and Intention Offloading. Replicating the findings from Gilbert (2015b), we found the former pattern for the Spontaneous Offloading Group (β = − 0.18, *p* = 0.03). For the Instructed Offloading Group, however, we found only a positive, non-significant regression weight (β = 0.05, *p* = 0.54).

The path analysis moreover revealed that participants had good confidence *resolution*, or accurate insight into how well they would perform. This is reflected in the significant positive path weights predicting Phase 1 performance from Phase 1 prediction (A; Instructed Offloading Group: β = 0.31, *p* < 0.001; Spontaneous Offloading Group: β = 0.31, *p* < 0.001) and Phase 2 performance from Phase 2 prediction (J; Instructed Offloading Group: β = 0.28, *p* < 0.001; Spontaneous Offloading Group: β = 0.30, *p* < 0.001). Our data furthermore supports the notion that participants learned over the course of the experiment: Performance during Phase 1 positively predicted prospective confidence (prediction) during Phase 2 in both groups (C; Instructed Offloading Group: β = 0.19, *p* < 0.001; Spontaneous Offloading Group: β = 0.36, *p* < 0.001). In other words, participants updated their Phase 2 prediction on the basis of their Phase 1 performance. Perhaps unsurprisingly, Phase 1 prediction positively predicted Phase 2 prediction (B; Instructed Offloading Group: β = 0.59, *p* < 0.001; Spontaneous Offloading Group: β = 0.51, *p* < 0.001), potentially reflecting that participants possessed an idiosyncratic confidence bias that was somewhat stable across the experiment. The same held for performance (G; Instructed Offloading Group: β = 0.54, *p* < 0.001; Spontaneous Offloading Group: β = 0.56, *p* < 0.001), meaning that participants who performed well within their group during the first phase of the task were likely to perform well within their group during the second phase of the task. Finally, we found a negative relationship between Phase 1 prediction and Phase 2 performance (I). The regression weight was significant for the Instructed Offloading Group (β = − 0.14, *p* < 0.05) but not for the Spontaneous Offloading Group (β = − 0.11, *p* = 0.12). Given that this effect was only significant in one of the groups in the present study, and neither of the experiments conducted by Gilbert (2015b), we consider that this result would require replication before considering it further.

In a final step, we tested whether the two groups differed in any of their path weights using multiple mixed-design Analysis of Covariance (ANCOVA) models. This analysis revealed only two differences in slopes between the two groups: Firstly, for the Spontaneous Offloading Group, the effect of Phase 1 performance on Phase 2 prediction was stronger (i.e. the slope was steeper, more positive) compared to the Instructed Offloading Group, as reflected in an interaction effect between Group and Phase 1 performance, *F* (1,357) = 7.4, *p* = 0.007. Secondly, for the Spontaneous Offloading Group, the effect of Phase 1 performance on Intention Offloading was stronger (and negative) compared to the Instructed Offloading Group, reflected in an interaction between Group and Phase 1 performance in the respective model, *F* (1,355) = 3.9, *p* = 0.048. However, neither of these effects survived Bonferroni correction for 10 path comparisons (α = 0.005)

## Discussion

In the present study, we investigated participants’ ability to remember delayed intentions in a task that allowed the possibility of setting external reminders. We found that participants set reminders not only when explicitly instructed to do so but also when they generated this strategy themselves. Critically, we found that both types of offloading were modulated by participants’ metacognition; more specifically, how confident they felt in their own memory performance. When people felt less confident, they were more likely to set reminders, independent of how well they actually performed. This was true both when the reminder-setting strategy was explicitly instructed and when it had to be generated spontaneously. Our findings have implications for the study of cognitive offloading because they suggest that overlapping mechanisms may underlie both explicitly instructed and spontaneous offloading. This suggests that the conclusions drawn from previous studies in which participants were provided with specific offloading strategies might equally apply to more naturalistic settings. Unlike our earlier study (Gilbert, 2015b), there was no clear relationship between participants’ objective memory ability and their propensity to offload. This suggests that such a relationship may be dependent on the availability of direct performance feedback, which was provided in the earlier study but not here. It also suggests that metacognitive beliefs are a more reliable determinant of offloading behaviour than objective cognitive ability, seeing as metacognitive measures predicted offloading in both studies.

### Uses of metacognition: Managing internal and external resources

We argue that research on delayed intentions and prospective memory should broaden its focus to also study cognitive offloading, because the use of reminders constitutes an intentional, highly strategic decision to augment the mind. We therefore expect that the study of human reminder use should have important links to the literature on cognitive control. One such link is the finding that offloading is guided by metacognition, replicated in the present study. Assuming that metacognitive beliefs serve as a cue to guide people when to adopt compensatory strategies, such as offloading, furthermore matches findings from the metacognition literature: A wealth of studies has accrued that suggest that people possess good metacognitive insight into their decisions and memory (e.g. Baranski & Petrusic, 1994; Chua, Schacter, & Sperling, 2009; Fleming & Lau, 2014; Fleming, Weil, Nagy, Dolan, & Rees, 2010; Koriat, 1993; Rabbitt, 2002). Two main questions can be distinguished that have been addressed in this line of research. The first investigates how metacognitive knowledge is derived from internal signals (e.g. Boldt, de Gardelle, & Yeung, 2017; Fleming & Daw, 2017; Koriat, 1997; Pleskac & Busemeyer, 2010; Pouget, Drugowitsch, & Kepecs, 2016), whereas the second focusses on how such knowledge can then be used (metacognitive control; Nelson & Narens, 1990). Recent research has increasingly begun to focus on this latter aspect of metacognition, suggesting that metacognitive control plays a key role in communication (Bahrami et al., 2010; Shea et al., 2014), learning (Daniel & Pollmann, 2011; Guggenmos, Wilbertz, Hebart, & Sterzer, 2016; Metcalfe & Finn, 2008), allocation of attentional resources (Rummel & Meiser, 2013), the trade-off between exploration and exploitation (Boldt, Blundell, & De Martino, 2019), how humans approach future decisions (Boldt, Schiffer, Waszak, & Yeung, 2019) and cognitive control (Fernandez-Duque, Baird, & Posner, 2000). While most of these studies focus on situations in which an agent’s knowledge regarding the availability of internal, mental resources influences cognition, similar mechanisms likely hold for external resources as well as the interaction and integration of both internal and external resources. In fact, Weis and Wiese (2019) have recently shown that people hold (potentially erroneous) beliefs about the reliability of external devices and adjust their offloading accordingly. Relatedly, Risko and Dunn (2015) measured people’s predicted accuracy in recalling a string of letters for both an internal strategy (holding them in short-term memory) and an external strategy (writing them down on a piece of paper). Such prospective confidence in one’s own short-term memory was a reliable predictor of people’s self-reported offloading.

### Interventions to improve memory for ageing and brain injury

The focus of the present study lay not on the delayed intentions per se, but instead on the reminders that people set to successfully fulfil them in the future. Our design therefore stands in direct contrast to most prospective-memory designs, which prohibit the use of external aids to allow a more accurate measurement of people’s unaided memory abilities. Often, reminder-setting is even regarded as a sign of non-compliance with the task instructions. Here, we argue that while restricting the use of reminders is of course important in many contexts, future research also needs to investigate the use of cognitive offloading itself. There are, however, lines of research that have extensively studied the use of memory aids: Research on ageing or brain injury traditionally deals with memory impairments and how they can be ameliorated. Memory aids, such as reminders, play a key role in this line of research (for a review see Phillips et al., 2008; Sohlberg et al., 2007). Phillips et al. (2008); for example, review a range of studies that suggest that there is higher use of memory aids in older people, presumably because they have insight into their declining memory abilities. Lovelace and Twohig (1990) found a clear increase in the use of external aids with age, such as calendars, but no increase in the use of internal memory strategies, such as mental retracing. The increased use of reminders with age presumably affects naturalistic tasks more than highly controlled laboratory experiments, which may contribute to age improvements in such naturalistic tasks (‘age-PM paradox’; Maylor, 2008; Phillips et al., 2008). A common scenario studied in this line of research is remembering to take medication and how the use of different types of reminders can support this. Similarly, the literature on brain injuries has studied how external reminders can support patients in their everyday life (for a review see Thöne-Otto & Walther, 2008).

The findings from our present study suggest that in addition to training programmes aimed at improving memory function directly (Raskin & Sohlberg, 1996) or teaching neuropsychological patients or older adults the use of external aids (Fish et al., 2010), it might be beneficial to also train their metacognitive insight to optimise the use of those compensatory strategies. There are two key promising avenues for such intervention programmes. First, improving the (metacognitive) accuracy of people’s insight into their own memory skills should arguably lead to more optimal reminder use. A recent study from our group (Gilbert et al., in press) indeed found that providing metacognitive advice led to more optimal offloading decisions. A similar idea is currently being evaluated by a randomised control trial conducted by Fleming et al. (2017), who directly compare a metacognitive skills’ training with teaching patients with traumatic brain injury just the compensatory strategies without strengthening the insight into their own memory abilities. Targeting a metacognitive angle might be particularly valuable in this context: Patients have developed a sense of their own abilities and skills over the course of their lives which may not match the post-injury reality (see Knight, Harnett, & Titov, 2005). A second suggested avenue would address metacognitive bias, that is whether people have a tendency to over- or underconfidence relative to their actual performance. We find that lower confidence leads to more offloading. Could correcting possible overconfidence biases make people more prone to rely on external aids? Future studies should test whether confidence could be directly manipulated to increase or decrease reminder use where needed. Together, these different avenues could inform any future attempts to develop interventions based on metacognitive beliefs and their relationship with cognitive offloading. Such trainings could then be used in addition or alternative to conventional cognitive trainings.

### Limitations and future research

The main methodological challenge that our study faces was the development of a paradigm that did not explicitly mention the offloading strategy but still allowed participants to develop and use it without feeling like they were ‘cheating’. Here, we chose a cover story according to which the individual circles in the task had to be ‘charged’ prior to each trial. A first practice trial included moving the circles, thereby showing participants that it was possible to freely move the circles out of their numerical order. This manipulation was successful in that participants did indeed develop the spontaneous offloading strategy. However, we cannot rule out that some individuals might have developed the strategy but decided not to use it, afraid that this might go against the rules of the task or because they were afraid to be penalised later on. This would mean that our task actually underestimated the proportion of participants who self-generate the offloading strategy.

Moreover, cognitive offloading can be expected to be highly idiosyncratic—people might have preferences for certain types of reminders or technical aids. In addition, the chosen reminder type is likely task- or situation-specific. Our task might, therefore, not have grasped all forms of offloading. For instance, several participants reported that they repeated the instructions out loud to rehearse them throughout each trial. While we made a conscious decision to—in the interest of simplicity—only focus on circle placement as a reminder strategy, future studies will need to develop ways to measure offloading more broadly and to furthermore link it to instances of reminder-setting in everyday life. One additional factor that might apply to well-practiced real-world tasks, but not the unfamiliar task used here, is that individuals might base their offloading decisions on habit, or prior history of offloading in similar circumstances (see Scarampi & Gilbert, 2019, for direct evidence that prior use of an offloading strategy makes individuals more likely to offload in future). This might reduce the relative importance of metacognition as a determinant of offloading decisions, compared with the task that we used here. It should also be noted that real-world tasks might afford more opportunities for error feedback than the task we used here, which could be expected to increase the calibration between metacognitive judgements and objective accuracy.

## Conclusions

In summary, the present study takes a first step towards studying cognitive offloading in more naturalistic environments by comparing instructed to spontaneous offloading. We find that people self-generate an offloading strategy that helped them remember to fulfil delayed intentions. Importantly, both instructed and spontaneous reminder strategies were guided by metacognitive beliefs, suggesting that people closely track both internal and external resources carefully weighing between them to optimise their prospective-memory performance. Our findings suggest that improving people’s metacognitive insight into their own memory abilities could potentially optimise how and when people choose to cognitively offload, without the need to explicitly instruct those strategies. Metacognitive interventions thus constitute a promising avenue to improve fulfilment of delayed intentions.

## Supplementary information

**Additional file 1.** Supplementary Materials: Further methods and analyses.

## Data Availability

The datasets and scripts used during the current study are available from the corresponding author on reasonable request.

## Abbreviations

IQR: Interquartile range
MCQ: Metacognitions Questionnaire
