# Supplementary material for: Confidence guides spontaneous cognitive offloading
Source: Cogn Res Princ Implic. 2019 Dec 2;4:45. doi: 10.1186/s41235-019-0195-y (PMC6889107; doi:10.1186/s41235-019-0195-y)
Supplement: Supplementary file 1 — Additional file 1. Supplementary Materials: Further methods and analyses. [file 41235_2019_195_MOESM1_ESM.docx]

Additional file 1

# Confidence Guides Spontaneous Cognitive Offloading

Annika Boldt^1^ & Sam J Gilbert^1^

^1^Institute of Cognitive Neuroscience, University College London, UK

***Task Instructions***

Both groups:

*Now we will go on to the second half of the experiment. This will be almost the same, with one difference.*

*Previously, you could begin dragging the circles straight away. From now on, you will have to ‘charge’ them first. Unactivated circles will be displayed in white and in order to charge them, you will have to drag them over the battery placed in the middle of the square. Please note that you can charge the circles in any order you want.*

*Only after all ten circles have been charged will you be able continue with the task.*

Only Instructed Offloading Group:

*This also means that you will now be able to rearrange the circles on the screen if you like.*

*Some people find it helpful to drag the special circles near to the edge of the box to help them remember.*

*For example, if you had to remember to drag 5 to the left of the box, you could drag it near to there at the beginning, before you drag the 1. Then when you eventually got to 5, its location would remind you what to do. You should feel free to use this strategy if you like, but it's up to you.*

Both Groups after completing ‘battery’ practice block:

*Good work. To make things easier for you, you will from now on be able to charge the circles by just clicking on them once. The battery will therefore no longer be displayed on screen. You will still be able to move the circles around, but you don't have to.*

*As before, you can charge the circles in whatever order you want and you will not be able to continue with the task unless all the circles have been activated.*

***Bimodal offloading distributions***


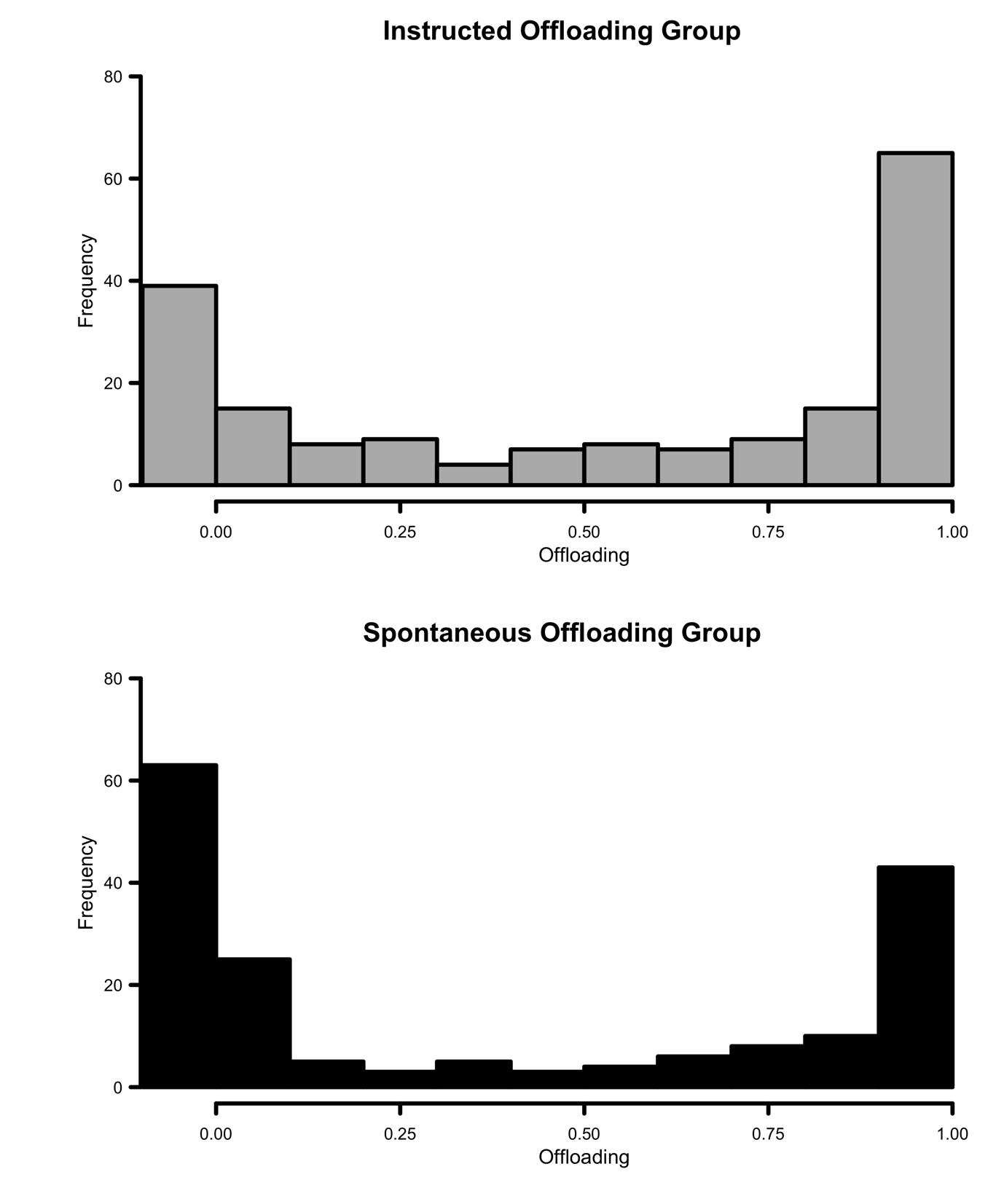


Figure S1: Histogram of average offloading across participants for the Instructed (top panel) and Spontaneous (bottom panel) Offloading Groups.

In the main text, we reported offloading proportions significantly different from zero for both groups and significantly more offloading in the Instructed Offloading Group, using parametric tests (one- and two-sample *t*-tests). Here, we repeat this analysis using non-parametric alternative tests. First, we used one-sample Wilcoxon signed-rank tests to determine whether both groups would show use of the offloading strategy. The Instructed Offloading Group had a median externalising proportion of 64.3%, which was reliably different from zero, *W* = 13569.0, *p* < 0.001. The same held for the Spontaneous Offloading Group, which had a considerably lower median externalising proportion of only 10.0%, but which was still significantly different from zero, *W* = 8785.5, *p* < 0.001. Second, the groups differed significantly in their use of reminders, as indicated by a Wilcoxon rank-sum test, *W* = 19482.0, *p* < 0.01. These findings therefore replicate the results of the parametric tests.

***Correlations of the variables reported in the path analysis***

Table S1: Correlations of the variables reported in the path analysis.

|  | **Offloading Group** | **Phase 1**  **Prediction** | **Phase 2**  **Prediction** | **Phase 1**  **Performance** | **Phase 2**  **Performance** | **Intention Offloading** |
| --- | --- | --- | --- | --- | --- | --- |
| **Phase 1**  **Prediction** | **Instructed** |  | *r* = 0.65  *p* < 0.001 | *r* = 0.31  *p* < 0.001 | *r* = 0.20  *p* < 0.01 | *r* = -0.05  *p* < 0.05 |
|  | **Spontaneous** |  | *r* = 0.62  *p* < 0.001 | *r* = 0.32  *p* < 0.001 | *r* = 0.23  *p* = 0.001 | *r* = -0.10  *p* = 0.18 |
| **Phase 2**  **Prediction** | **Instructed** |  |  | *r* = 0.36  *p* < 0.001 | *r* = 0.41  *p* < 0.001 | *r* = 0.12  *p* = 0.10 |
|  | **Spontaneous** |  |  | *r* = 0.49  *p* < 0.001 | *r* = 0.57  *p* < 0.001 | *r* = 0.18  *p* = 0.01 |
| **Phase 1**  **Performance** | **Instructed** |  |  |  | *r* = 0.61  *p* < 0.001 | *r* = 0.06  *p* = 0.43 |
|  | **Spontaneous** |  |  |  | *r* = 0.67  *p* < 0.001 | *r* = -0.01  *p* = 0.93 |
| **Phase 2**  **Performance** | **Instructed** |  |  |  |  | *r* = 0.30  *p* < 0.001 |
|  | **Spontaneous** |  |  |  |  | *r* = 0.27  *p* < 0.001 |
| **Intention Offloading** | **Instructed** |  |  |  |  |  |
|  | **Spontaneous** |  |  |  |  |  |
